# Suppression of FOXO1 activity by SIRT1-mediated deacetylation weakening the intratumoral androgen autocrine function in glioblastoma

**DOI:** 10.1038/s41417-025-00880-1

**Published:** 2025-03-12

**Authors:** Yuanchi Cheng, Zhijun Xiao, Weijia Cai, Ting Zhou, Zhen Yang

**Affiliations:** 1 Department of Neurosurgery, Shanghai University of Medicine & Health Science Affiliated Sixth People’s Hospital South Campus, Shanghai, 201499 China; 2 Department of Pharmacy, Shanghai University of Medicine & Health Science Affiliated Sixth People’s Hospital South Campus, Shanghai, 201499 China; 3 Department of Central Laboratory, Shanghai University of Medicine & Health Science Affiliated Sixth People’s Hospital South Campus, Shanghai, 201499 China

**Keywords:** Cancer microenvironment, Cancer therapy

## Abstract

Elevated levels of androgens in the brain accelerate tumor progression in patients with glioblastoma (GBM). Despite current research efforts concentrating on decreasing peripheral androgens to improve GBM prognosis, results have not met expectations. Herein, we aim to elucidate the source of increased androgen levels in the brains of GBM patients and investigate whether lowering it can improve the prognosis of GBM patients. The Elisa was employed to measure androgen levels. The effects of androgens on U87 cells were evaluated using CCK-8 assays, clone formation assays, wound healing assays, and migration/invasion assays. RNA sequencing, RT-qPCR and Western blotting were performed to assess the expression levels of steroid enzymes, tumor drug resistance, Sirt1, FOXO1genes and proteins. Co-immunoprecipitation (Co-IP) assays were conducted to investigate the interactions and acetylation levels between Sirt1 and FOXO1. Lentiviral transfection was utilized to establish stable cell lines. Furthermore, an in vivo murine subcutaneous tumor model was established to further confirm the role of Sirt1 in tumor progression. We found androgen levels in the cerebrospinal fluid of GBM patients were higher than in the periphery, contrasting with healthy individuals. Additionally, the steroid enzymes in GBM cells were upregulated. Reducing peripheral androgens compensatorily enhances GBM androgen synthesis capacity (CYP17A1, CYP11A1, SRD5A2) and chemo-resistance (ABCB11, BIRC3, FGF2, NRG1), while the levels of androgens in the brain remain consistently high. The above results indicate that the increased androgens in the brain of GBM patients are self-secreted. Further investigations demonstrate that the transcription factor FOXO1 in GBM is regulated by silent information regulator 1 (Sirt1) through deacetylation, leading to enhanced androgen synthesis capacity in vivo and in vitro. Overexpressing Sirt1 significantly lowers brain androgen levels and delays tumor progression in mouse models. Compared to conventional finasteran therapy, the targeted-Sirt1 results in lower brain androgen levels and smaller tumor volumes. Our findings provide evidence that the elevated androgens in the brain of GBM patients came from tumor autocrine. Overexpression of Sirt1 reduces FOXO1 acetylation, lowers androgen synthesis enzyme levels, and effectively decreases brain androgen levels, thereby delaying tumor progression.

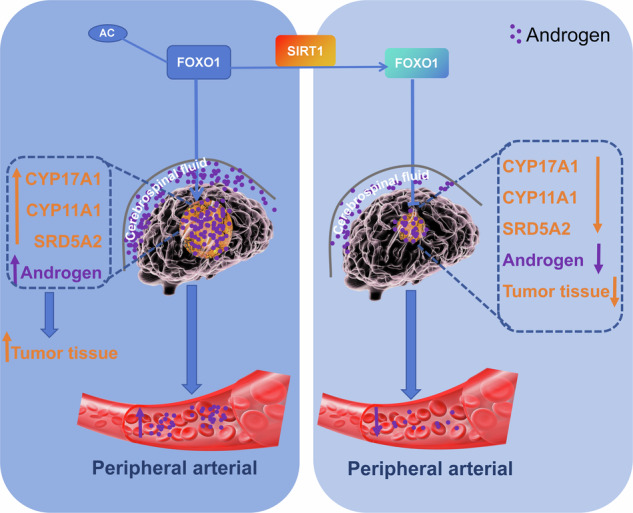

## Introduction

Glioblastoma (GBM) is an aggressive brain tumor with a dismal prognosis, characterized by a 5-year survival rate of only 5% despite intensive treatment efforts [1, 2]. Research suggests that androgen, a male hormone, and its receptor play a significant role in GBM progression and contribute to chemotherapy resistance in GBM cells [3]. Notably, males tend to experience higher morbidity and faster disease progression compared to females [4–6]. GBM patients often show elevated serum testosterone levels, which normalize after surgery [7]. Increased androgen receptor (AR) expression in GBM tissue correlates with higher pathological grading [8–10]. Gender differences in GBM are further highlighted by differences in cellular, molecular, and imaging characteristics [7, 11]. These findings underscore the significant involvement of androgens in GBM development, though the exact mechanisms remain elusive.

The transcriptional activity of Forkhead box O (FOXO) family members is intricately governed by a multifaceted signaling network that reacts to environmental stimuli through various posttranslational modifications, including phosphorylation, acetylation, ubiquitination, and methylation [12–14]. FOXO1, as a representative transcription factor within the FoxO family, primarily relies on its phosphorylation and nuclear localization for its activity [15–17]. Additionally, acetylation and deacetylation also play pivotal roles in modulating the transcriptional activity of FOXO proteins. For example, p300 enhances FOXO1-mediated transcription by directly acetylating FOXO1 in its carboxyl-terminal region, while silent information regulator 1 (Sirt1) serve as deacetylases for FOXO1, thereby contributing to the regulation of its activity [18, 19]. The activity of FOXO1 is enhanced in various hormone-dependent tumors [20].

Sirt1, a NAD-dependent deacetylase, is involved in the deacetylation of FOXO, NF-κB, and PGC-1α, playing a crucial role in cell proliferation and differentiation [14, 21–23]. Initially identified as a tumor promoter due to its capability to downregulate multiple anticancer factors, recent evidence suggests a dual role for Sirt1, also acting as a tumor suppressor [24–26]. Decreased expression of Sirt1 is observed in various cancers such as GBM, bladder cancer, prostate cancer, ovarian cancer, and breast cancer, where it exerts its tumor-suppressive effects [27–29]. In vitro studies have demonstrated that Sirt1 suppresses the activity of Bax, FOXO, and retinoblastoma [30]. In addition, the FOXO-1/Sirt1 axis plays a role in the synthesis of the testes [31]. In androgen-deprived prostate cancer, the activation of the FOXO-1/Sirt1 axis promotes tumor progression [32, 33]. The androgen-mediated Sirt1-FOXO axis may be involved in the progression of tumors, but its role in GBM is still unclear.

In our current work, we have uncovered the presence of an autocrine androgen pathway activation within GBM cells. Interestingly, reducing exogenous androgen didn’t hinder tumor progression but instead stimulated an increase in intracellular androgen synthesis capacity, leading to heightened resistance to chemotherapy. Sirt1 inhibits the autocrine androgen process in the brain by deacetylating FOXO1. These findings suggest that GBM cells may possess the ability to produce androgen internally. Additionally, we demonstrate that Sirt1 plays a vital role in suppressing the transcriptional activity of FOXO1 through deacetylation, leading to decreased androgen secretion and ultimately suppressing tumor progression in GBM.

In our current work, we found elevated of androgen level in the cerebrospinal fluid of GBM patients, even androgen levels were higher than periphery, contrary to healthy people. Then we clarified the promoting effect of androgens on GBM cells. Interestingly, reducing exogenous androgen didn’t hinder tumor progression but instead stimulated an increase in intracellular androgen synthesis capacity, leading to heightened resistance to chemotherapy. We further revealed that GBM cells may possess the ability to produce androgen internally. Morever Sirt1 inhibits the autocrine androgen process in the brain by deacetylating FOXO1. Importantly, the therapeutic effect of targeting-Sirt1 is superior to conventional finasteride therapy. Taken together, our findings suggest that the elevated androgens in the brains of GBM patients originate from tumor autocrine. Suppression of FOXO1 activity through SIRT1-mediated deacetylation weakens the intratumoral androgen autocrine function in GBM. Therefore, targeting Sirt1 can provide novel ideas for the treatment of GBM.

## Materials and methods

### Reagents

All the antibodies used are listed in Table S1. SIRT1-specific agonist SRT2104(s7792) and SIRT1-specific antagonist Selisistat(s1541) were from Selleckchem (Houston, TX, USA). Plasmids for Sirt1 overexpression, FOXO1-siRNA were purchased from GeneChem Biotechnology Company (Shanghai, China).

### Patient tissue specimens and cell lines

Prior to conducting this study, ethical approval was obtained from the Institutional Review Board of Shanghai Fengxian District Central Hospital(2020-KY-07). Written informed consent was acquired from all human subjects involved in the study. Between 2020 and 2022, a total of 133 specimens of glioma tissues were procured from patients undergoing surgical excision procedures at the Department of Neurosurgery, Fengxian District Central Hospital in Shanghai. These specimens were categorized according to the 2016 World Health Organization Consensus Classification, encompassing stages ranging from I to IV, to ensure standardized classification. This study included patients diagnosed with glioma, aged ≥18 years, who provided informed consent. Participants were required to have not received any form of hormone therapy prior to sample collection and to be free of other malignancies or severe comorbidities. Exclusion criteria comprised recent infections, inflammatory conditions, and patients with significant cognitive impairments. CSF and venous blood were sampled at the same time during the first week after the patient’s diagnosis. Tumor tissue was obtained during the surgery. The sample size was estimated based on previously [34]. Additionally, the human glioblastoma cell line U87, human lung adenocarcinoma cell line A549 and human embryonic kidney cell line 293 T were sourced from the Shanghai Cell Bank, affiliated with the Chinese Academy of Sciences in Shanghai, China. All cells were authenticated and tested for mycoplasma contamination. These cell lines were cultured in DMEM medium supplemented with 10% fetal bovine serum and 1% penicillin/streptomycin. Incubation took place at a temperature of 37 °C in an environment composed of 5% CO_2_ and 95% humidified air.

### Nude mouse intracranial model

#### Mice

Female nude mice aged 4-6 weeks were purchased from Shanghai JSJ Laboratory Animal Co., Ltd. (Animal Quality Certificate: 20230004050501). This study was approved by the Institutional Animal Care and Use Committee of Shanghai Sixth People’s Hospital(2023-0379). None of the animals were excluded from the study. After a 7-day adaptation period in the animal laboratory with appropriate feeding, all animals were prepared for experimentation. The U87 cells were suspended in PBS and adjusted to a density of 2×10^8^ cells/mL. The mice were anesthetized and positioned on a stereotactic apparatus. Using precise coordinates (1 mm anterior to the bregma and 2-3 mm lateral to the midline, while avoiding blood vessels), a cranial hole was carefully drilled. The injection needle was then inserted to a depth of approximately 3.5 mm, and a volume of 5 μL of cell suspension was slowly injected into the mouse brain parenchyma in a vertical direction (at a concentration of 1×10^6^ cells per mouse). Following completion of the injection, the needle remained in place for 5 minutes before being retracted slowly. Magnetic Resonance Imaging(MRI) scans were conducted 10 days post-injection completion.The sample size for each experiment was estimated based on previously [35]. In this study, All animals were randomly assigned to 4 groups(n = 6) and were euthanized by cervical dislocation for tumor, CSF, blood sample collections. Blinding was performed during data analysis.

### Cell Viability Assay by CCK-8

In the experiment investigating the effects of testosterone on U87 cell proliferation, a gradient of testosterone concentrations was set: 1, 10, 20, and 40μmol/L. The final concentration selected for the experiment was 20μmol/L and representing the high-level androgen group. The experiment was then divided into three groups: control group, charcoal-stripped fetal bovine serum group (CFBS), and high-level androgen group (Double Hydrogen Testosterone (DHT), 20μmol/L). U87 cells (5000 cells per well) were cultured in a 96-well plate for 24 h, 48 h, and 72 h, respectively. Cell viability was assessed using CCK-8 assay. The OD values at 450 nm were measured and recorded using the GloMax Multi+ machine (Promega, E8032, USA).

### Protein Expression Analysis

Tumor tissues and cells underwent ultrasound treatment in ice-cold lysis buffer. Following centrifugation, the protein concentration in each sample was determined by employing the BCA protein quantification kit. Subsequently, SDS-PAGE was conducted to separate and transfer total proteins(20 μg) from each sample onto a PVDF membrane. This membrane was blocked using a 4 h incubation in a solution comprising 5% skim milk in Tris-buffered saline with Tween 20 (TBST), followed by an overnight incubation at 4 °C with primary antibodies. All antibodies were suitably diluted in 5% skim milk-containing TBST buffer. Specifically, CYP17A1, CYP11A1, SRD5A, ABCB11, and NRG1 were diluted at ratios of 1:1000, whereas BIRC3, FGF2 and β-actin were diluted at ratios of 1:2000. The membrane was subjected to three 10 minute washes with TBST buffer, succeeded by a 2 h incubation with secondary antibodies at room temperature. Ultimately, the visual analysis of the membrane was performed using an ECL detection reagent, and the subsequent data analysis was carried out utilizing ImageJ software.

### Real-time PCR analysis

Total RNA was isolated from cultured cells with TRIzol reagent and subsequently reverse-transcribed into complementary DNA (cDNA), Real-time PCR was carried out and the relative expression levels of the target genes were determined by the 2 − ΔΔCt method. The primers for qRT-PCR: GAPDH-F: GGAGCGAGATCCCTCCAAAAT, GAPDH-R: GGCTGTTGTCATACTTCTCATGG.FOXO1-F:CATTGCGGAAAGAGAGAGCC, FOXO1-R: AGGGGTTGCTGCTGAAATTG

### Immunoprecipitation

Extracts for immunoprecipitation (IP) were prepared using cell lysis buffer for western blotting and IP blotting, containing a protease inhibitor. The extracts were incubated with magnetic beads-conjugated antibody at 4 °C overnight on a rotator. The samples were analyzed by SDS-PAGE, followed by western blotting using the appropriate antibodies.

### Hematoxylin and eosin (H&E) staining

The tumor tissues were cut into thin sections with a thickness of 10 μm. These sections were then subjected to staining using H&E procedures.

### Wound healing assays

Cells were seeded in a 6-well plate and cultured until they reached approximately 90% confluence. Artificial wounds were created using a plastic pipette tip, and the wound area was captured at 0 h, 4 h, 8 h, and 12 h time points.

### Cell Migration Assay

Cells were seeded in a 6-well plate and treated for 72 h. After that, the cells were collected and resuspended in 0.1 ml of DMEM (5×10^4^ cells/well) in the transwell upper chambers. The culture medium used was DMEM. The bottom chamber was filled with 0.6 ml of DMEM with 10% FBS as a chemoattractant. After 12 h, non-migratory cells were carefully removed using a cotton swab. Subsequently, the cells were stained with crystal violet for 10 minutes.

### Colony Formation

The cells were seeded in 6-wells plates at the density of 1000 cells per well. and incubated at 37 °C in a 5% CO_2_ humidified atmosphere for 1 week, and the colonies with more than 50 cells were scored. After the desired time, the cells were fixed using 4% paraformaldehyde and dyed by crystal violet. The number and size of the colonies were then quantified.

### Cell invasion Assay

U87 cells were seeded in a serum-free medium at the density of 1000 cells per upper chamber of each pre-coated insert. The lower chamber was filled with 0.6 mL of 10% FBS as a chemoattractant. The transwell plates were incubated at 37 °C with 5% CO_2_ in a humidified incubator for 12 h. Then, non-invading cells on the upper side of the insert were gently removed. Invaded cells that had penetrated the Matrigel® layer and migrated through the pores to the lower side were fixed with 4% paraformaldehyde for 15 minutes. Subsequently, the cells were stained with crystal violet for 10 minutes.

### RNA Sequencing

The Trizol reagent was utilized to isolate total RNA from tumor tissue following the provided protocol. Subsequently, libraries were generated, and sequencing was performed to obtain 150 bp paired-end reads. A total of approximately 63 G reads and 430 M clean reads were acquired across all samples. The cleanliness of the reads was assessed using cufflinks, while read counts were determined by HTSeq-count. Statistical significance was determined based on a threshold of P < 0.05 and a fold change >2 or <0.5.

### Lentiviral transfection

Cells were seeded in 6-well plates at a density of 2×10^5^ cells. After 24 h, the cells were infected with a polybrene-containing culture medium (6ug/ml), MOI = 30. Following a 24 h infection period, the cells were transferred to fresh complete medium and cultured further. At 72 h post-infection, the mRNA level of Sirt1 was assessed, showing an infection efficiency of approximately 80%. Subsequently, puromycin (puro) was added to each well to monitor cell growth. After approximately 48 h, the viability of cells in the control group was observed. If around 90% of the cells in the control group exhibited cell death, puromycin was removed, and the cells were switched to fresh medium for subsequent cultivation and passaging. Stable clone selection was then conducted. The Sirt1 overexpression cell line (pc-Sirt1) has been successfully constructed.

### Statistical analysis

Statistical analyses were conducted using either SPSS 22.0 or GraphPad Prism 9.5 software. All in vitro experiments were replicated three times. The data are expressed as the mean ± SD. The data were analyzed with an unpaired Student’s t test without assuming equal variance and one-way analysis of variance. One-way analysis of variance (ANOVA) with a two-tailed test was used for intergroup comparisons. *P* value < 0.05 was considered statistically significant.

## Results

### Testosterone levels and receptor are up-regulated and associated with a poor prognosis in GBM

**Fig. 1:**
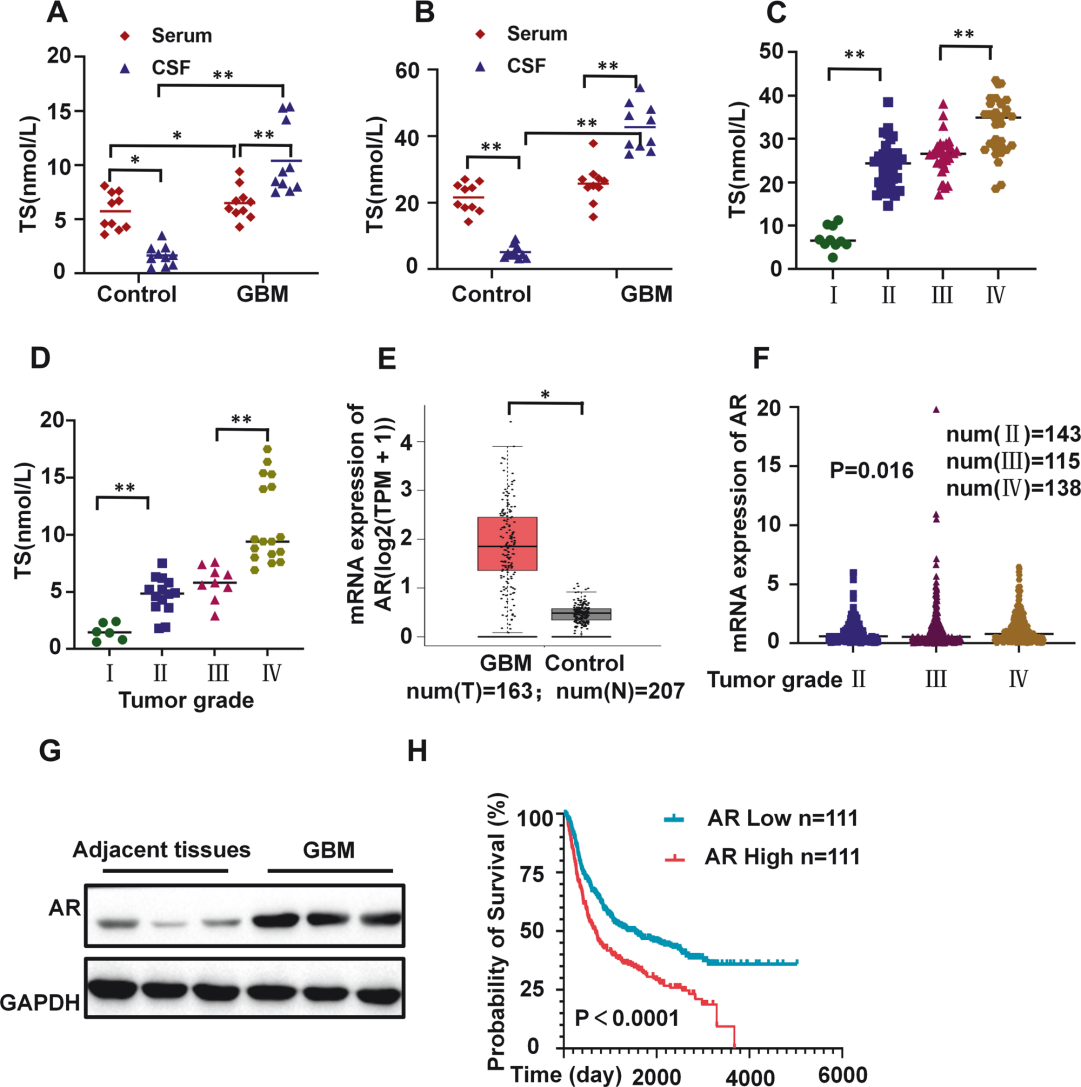
Effects of Androgen Hormone and Receptor Levels on GBM Patient Survival. **A** Serum and CSF testosterone (TS) levels in female patients with traumatic brain injury or GBM (n = 10). **B** Serum and CSF testosterone levels in male patients with traumatic brain injury or GBM (*n* = 10). **C** Testosterone levels in the CSF of male patients with GBM at various grades(num(I)=10; num(II)=28; num(III)=20; num(IV)=29). **D** Testosterone levels in the CSF of female patients with GBM at various grades(num(I)=6; num(II)=14; num(III)=9; num(IV)=17). **E** Comparison of AR expression levels in GBM patients with control group from the TCGA database(num(T) = 163；num(N) = 207). **F** Comparison of AR expression levels in GBM patients with different grades from the TCGA database(num(II)=143; num(III)=115; num(IV)=138). **G** Protein expression levels of AR in GBM and peritumoral tissue of patients(n = 3). **H** The Kaplan-Meier curve shows survival differences between GBM patients with high and low AR expression from the Chinese Glioma Genome Atlas(CGGA) database(n = 111). **P <0.01, *P <0.05.

To investigate the potential involvement of androgen in the regulation of GBM progression, the levels of testosterone in the cerebrospinal fluid (CSF) and peripheral blood of GBM patients of different genders were assessed. Comparing testosterone levels in CSF and peripheral blood between traumatic brain injury patients and those with GBM, CSF testosterone levels significantly elevated in both male and female GBM patients. (Fig. 1A & B). To determine the potential association between testosterone levels and glioma grades, we re-collected CSF samples from patients with different grades of glioma(I, II, III, IV). Higher tumor grades correlated with increased CSF testosterone levels in both genders (Fig. 1C & D).

The mRNA expression of AR was significantly upregulated in GBM tissues relative to that in normal brain tissues. The AR expression level positively correlates with increasing tumor grade. Western blotting confirmed that AR expression was upregulated in GBM tissue compared with peritumoral tissue. (Fig. 1E~G). Notably, GBM patients with higher expression of androgen receptors had shorter survival periods (Fig. 1H). These findings suggest a potential link between elevated CSF testosterone levels and poor prognosis in GBM patients.

### Androgen levels in CSF are elevated possibly due to autocrine secretion by tumor cells

Considering that androgen may be a biomarker for GBM prognosis, the impact of androgen on the progression of GBM were assessed in vitro. We tested various concentrations of dihydrotestosterone (DHT) on U87 cell proliferation and found 20 μmol/L to be the optimal dose (Figure S1A). Our findings revealed that elevated DHT levels heightened tumor invasion, migration, and colony formation, while exerting minimal effects on cell proliferation. Conversely, removal of the hormone led to a significant inhibition of tumor cell proliferation, invasion, migration, and colony formation (Figure S1B~F). These results further corroborate the notion that elevated androgen levels facilitate tumor progression, whereas lower levels act to suppress it.

**Fig. 2:**
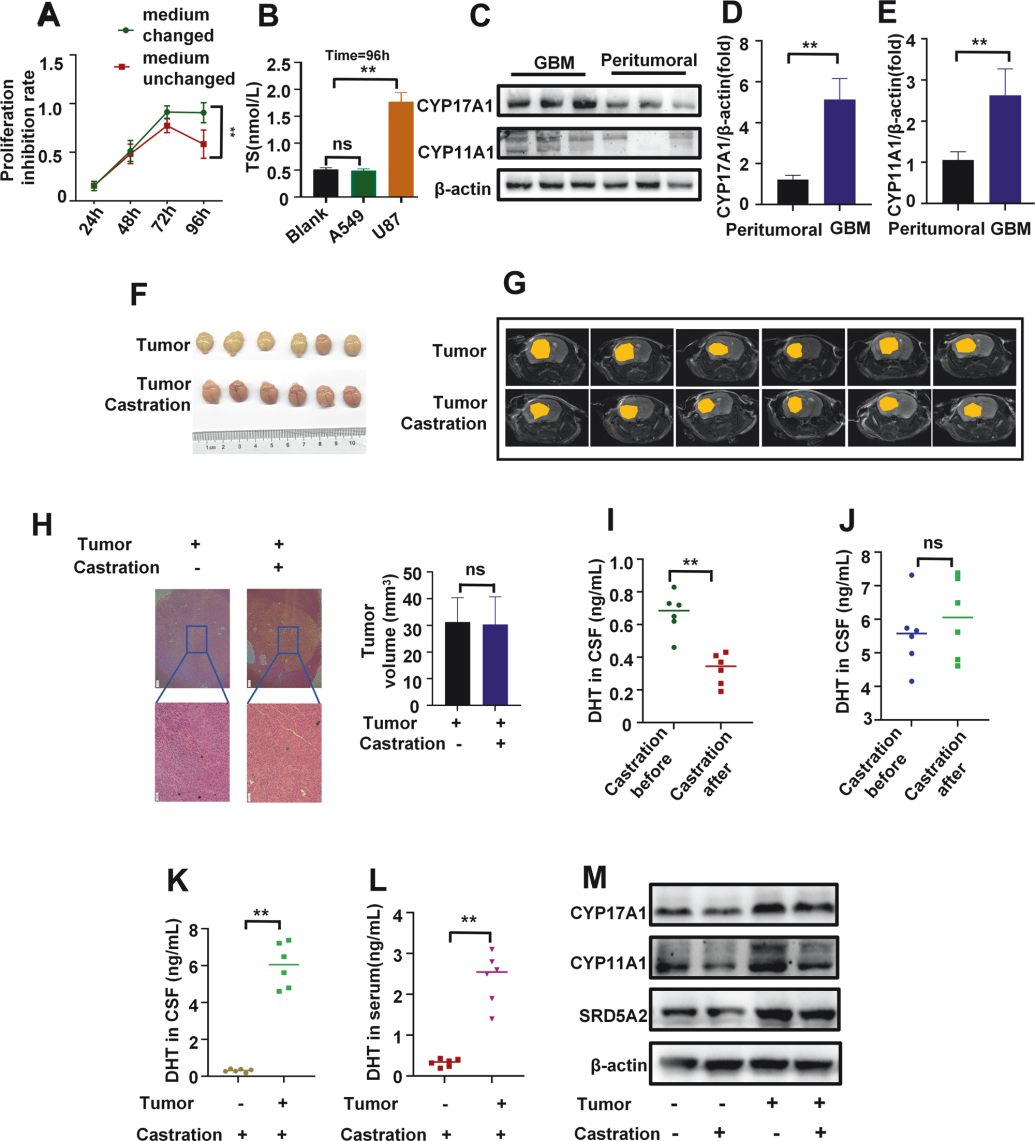
GBM cells can secrete androgen. **A** Proliferation curve of U87 cells under charcoal-stripped fetal bovine serum(CFBS) culture (24 h medium change vs continuous medium retention, *n* = 3). **B** Androgen concentration in a blank control medium, the culture medium of A549(negative control) and U87 cells cultured for 96 h under CFBS culture (continuous medium retention, *n* = 3). **C** Protein expression levels of CYP17A1 and CYP11A1 in GBM and peritumoral tissue of patients(n = 3). **D, E** Relative mRNA expression levels of CYP17A1 (**D**) and CYP11A1 (**E**) in GBM and peritumoral tissue of patients (*n* = 3). **F**, **G** The tumor images (**F**) and Magnetic Resonance Imaging (MRI) images(G) of mice in the castrated and non-castrated groups (*n* = 6). **H** H&E images and tumor volume of mice in the castrated and non-castrated groups(*n* = 3), scale bars=200μm. **I, J** Levels of androgen in CSF of mice before and after castration in the control group (**I**) and tumor group(**J**) (*n* = 6). (K&L) Levels of androgen in CSF(**K)** and peripheral blood serum (**L**) in the castration group and tumor castration group of mice (*n* = 6). **M** Steroid enzyme protein expression in the tumor group and castration group of mice (*n* = 3). **P <0.01.

While hormone depletion exhibited inhibitory effects on progression of GBM cells, reducing testosterone levels did not significantly improve therapeutic outcomes in clinical settings. In order to gain deeper insights into the impact of hormone depletion on GBM progression, we extended the duration of our experiments. We observed that the inhibitory effect of hormone deprivation on tumor cells gradually diminished after 72 hours (Fig. 2A). When comparing these observations with non-hormone-dependent A549 lung adenocarcinoma cells, we noted that after 96 hours of continuous culture without medium replacement, testosterone was detected in the U87 cell culture medium, but not in A549 (Fig. 2B). In GBM patients, we observed a significant increase in level of androgen in both peripheral blood and CSF. Meanwhile, the levels of androgens in CSF are higher than in peripheral blood, which is in contrast to healthy individuals(Fig. 1A & B). When comparing tumor tissues with peritumoral tissues in patients, we observed a notable upregulation of androgen synthesis enzymes, specifically CYP17A1 and CYP11A1 (Fig. 2C~E). All of these findings suggest that U87 cells may synthesize testosterone.

Testosterone is primarily produced by testicular Leydig cells in males. Castration of GBM mice was performed to determine the source of increased androgen levels. In animal experiments involving tumor-bearing groups and tumor-bearing castrated groups, we observed that castration had no effect on tumor progression (Fig. 2F~H). While castration significantly reduced testosterone levels in the serum of the control group, it did not affect the tumor group (Fig. 2I & J). Interestingly, we found that the tumor-bearing castrated group exhibited significantly higher testosterone levels in both serum and CSF compared to the castrated-only group (Fig. 2K & L). Similarly, the tumor group displayed a notable increase in steroid enzymes compared to the control group, and this increase remained unaffected by castration (Fig. 2M). These findings suggest that the tumor itself is the primary factor contributing to elevated testosterone levels. Taken together, these results strongly indicate that high levels of testosterone in the CSF originate from autocrine secretion by GBM cells.

### Enhanced androgen synthesis and drug resistance genes upregulation in GBM under hormone deprivation

**Fig. 3:**
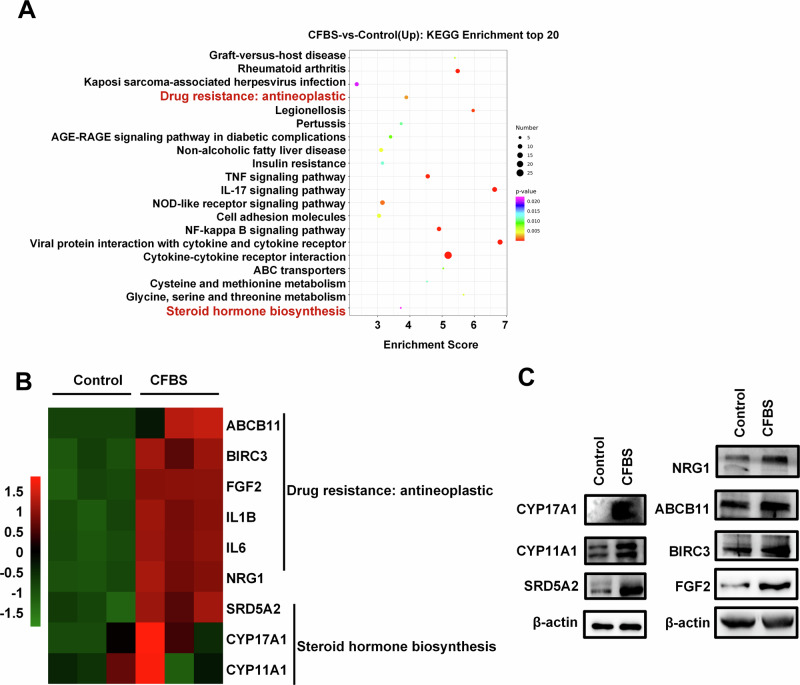
Effects of hormone deprivation on GBM cell. **A** KEGG enrichment analysis of differentially expressed genes after U87 cells underwent a 96-hour culture in de-hormone medium and normal medium (*n* = 3). **B** RNA-seq data between de-hormone medium and normal medium groups of U87 cells (*n* = 3). **C** Effects of CFBS treatment for 96 h on steroid enzyme and drug resistance protein in U87 cells (*n* = 3).

To further investigate the molecular mechanism underlying testosterone release in U87 cells, transcriptome sequencing analysis was subsequently performed. U87 cells underwent a 96-hour culture in both de-hormone medium and normal medium, followed by transcriptome sequencing. The results indicated that compared to cells cultured in normal medium, those in de-hormone medium exhibited an upregulation of steroid enzymes CYP17A1, CYP11A1, and SRD5A2, which led to a compensatory enhancement of androgen synthesis capability. Additionally, hormone deprivation triggered an upregulation of tumor drug resistance genes ABCB11, BIRC3, FGF2 and NRG1(Fig. 3). This finding suggests that solely reducing peripheral androgen levels might not effectively lower androgen levels or impede tumor progression.

### FOXO1 protein level modification promotes GBM progression

**Fig. 4:**
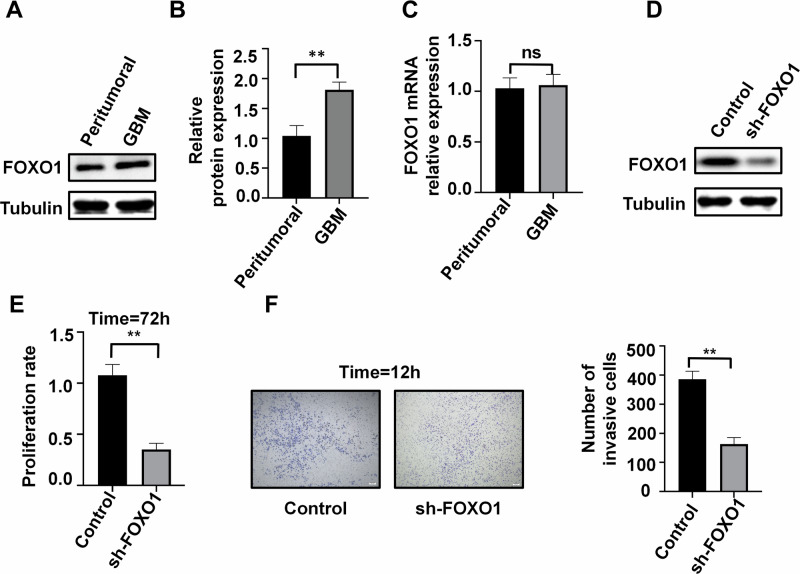
Effects of FOXO1 on GBM proliferation and invasion ability. **A, B** Protein expression of FOXO1 in tumor and peritumoral tissue of patients (*n* = 3). **C** mRNA expression of FOXO1 in tumor and peritumoral tissue of patients (*n* = 3). **D** Silencing of FOXO1 protein in U87 cells (*n* = 3). **E, F** Effects of FOXO1 silencing on cell proliferation (T = 72 h, E) and cell invasion (T = 12 h, F) in U87 cells (*n* = 3), scale bars=200μm. **P < 0.01.

Since FOXO1 plays a role in regulating androgen synthesis, androgen receptor activity, and spermatogenesis [36, 37], we compared the mRNA and protein expression levels of FOXO1 in GBM and normal tissues. Surprisingly, compared to peritumoral tissue, the protein expression level of FOXO1 in tumor tissue was significantly elevated, while there was no significant difference in mRNA expression levels between the two groups (Fig. 4A~C). This suggests that FOXO1 may undergo protein modification. We further silenced FOXO1 using shRNA and found that inhibition of FOXO1 expression reduces the proliferation and invasion of U87 cells (Fig. 4D~F).

### Sirt1 physically interacts with and acetylates FOXO1, down-regulates DHT synthesis in GBM cells

**Fig. 5:**
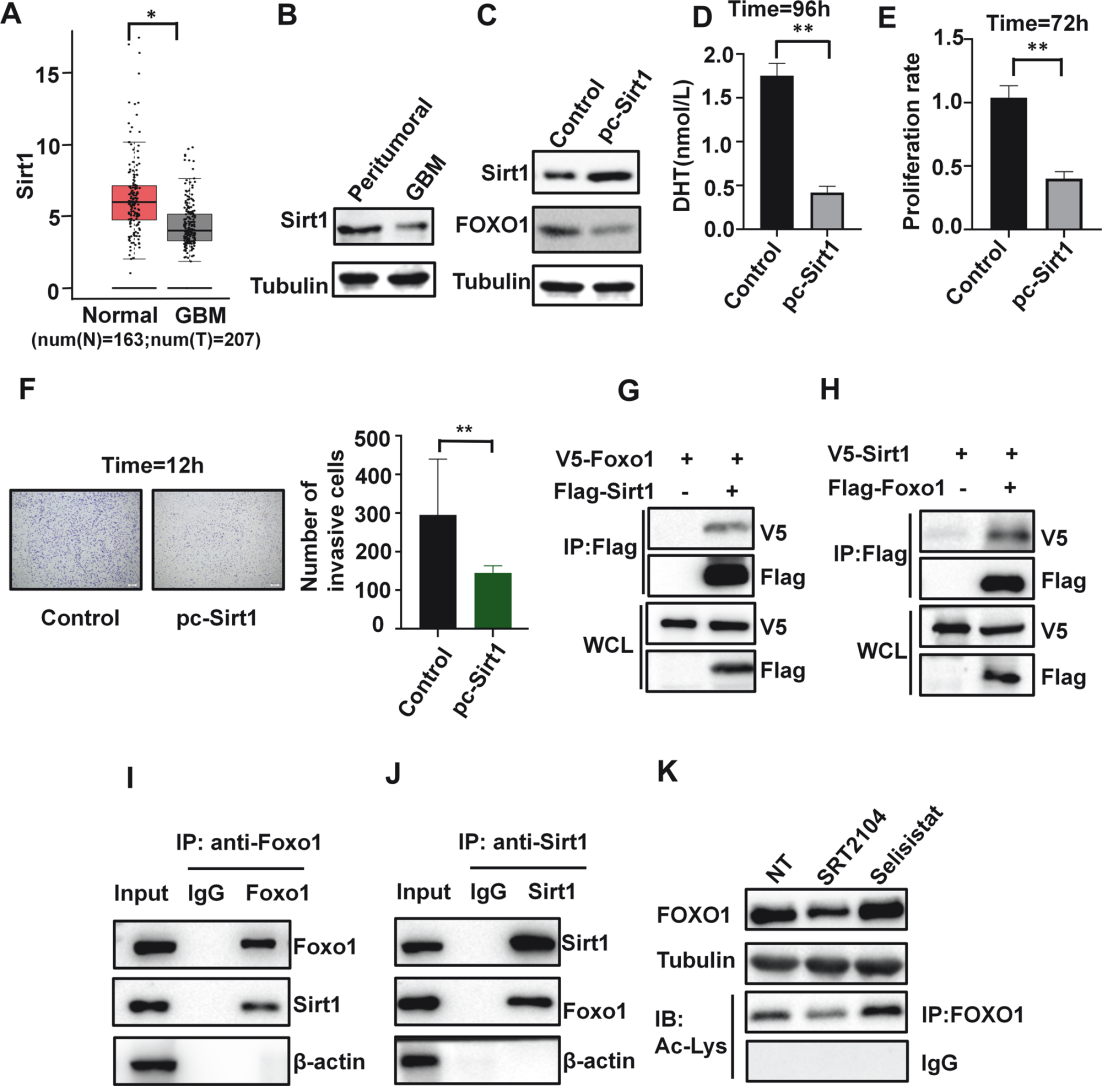
Sirt1 physically interacts with FOXO1 and regulates the FOXO1 acetylation. **A** Expression of Sirt1 in tumor and normal brain tissue in the TCGA database(num(N) = 163;num(T) = 207). **B** Protein expression of Sirt1 in tumor and peritumoral tissue (*n* = 3). **C** Western blotting showing FOXO1 protein in U87 cells transfected with control vector or pc-Sirt1 (*n* = 3). **D** Effects of Sirt1 overexpression on DHT synthesis in U87 cells(*n* = 3). **E** Effects of Sirt1 overexpression on cell proliferation (*n* = 3). **F** Effects of Sirt1 overexpression on cell invasion (*n* = 3), scale bars=200μm. **G, H** The 293 T cells were transfected with plasmids, and subsequently subjected to immunoprecipitation using an antibody targeting the Flag tag, then analyzed through immunoblotting using antibodies against both the Flag and V5 tags (*n* = 3). **I, J** To explore the inherent interaction between Sirt1 and FOXO1, protein extracts from U87 cells were immunoprecipitated using specific antibodies against FOXO1 (**I)** and Sirt1 (**J**), followed by analysis of the precipitated samples using immunoblotting (*n* = 3). **K** FOXO1 expression in the presence of Sirt1 activator SRT2104 or Sirt1 inhibitor Selisistat. FOXO1 proteins were deacetylated by Sirt1. The global acetylation level of FOXO1were detected by immunoprecipitation, by treating U87 cells with anti-Ac-Lys antibodies (*n* = 3). *P < 0.05, **P < 0.01.

Based on analysis using the public GEPIA dataset (gepia2.cancer-pku.cn), which sources data from TCGA and GTEx, we found that Sirt1 is significantly downregulated in GBM tissue compared to normal tissue (Fig. 5A). Additionally, in our in-house clinical samples, we also found that the protein expression levels of Sirt1 in GBM tissue were lower than those in peritumoral tissue (Fig. 5B). The overexpression of Sirt1 reduces the protein level of FOXO1 (Fig. 5C), resulting in the inhibition of androgen production in U87 cells(Fig. 5D), consequently suppressing tumor cell proliferation and invasion (Fig. 5E & F).

To investigate the interaction between Sirt1 and FOXO1, we performed co-transfection of V5-tagged and FLAG-tagged plasmids in HEK293T cells. Co-immunoprecipitation (co-IP) experiments using an anti-FLAG antibody revealed a significant interaction between Sirt1 and FOXO1 (Fig. 5G & H). Additionally, the interaction between endogenous Sirt1 and FOXO1 was confirmed in U87 cells (Fig. 5I & J). It is worth noting that Sirt1 functions as an enzyme that deacetylates histone proteins and other substrates [38], therefore, it is necessary to verify whether Sirt1 regulates FOXO1 acetylation. Stimulated with the Sirt1-specific activator SRT2104 or the Sirt1-specific antagonist Selisistat in U87 cells, proteinomic and acetylomic analysis revealed that Sirt1 significantly reduced the acetylation level and protein expression of FOXO1 (Fig. 5K).

### Sirt1 overexpression suppresses tumor growth and reduces androgen levels in CSF

**Fig. 6:**
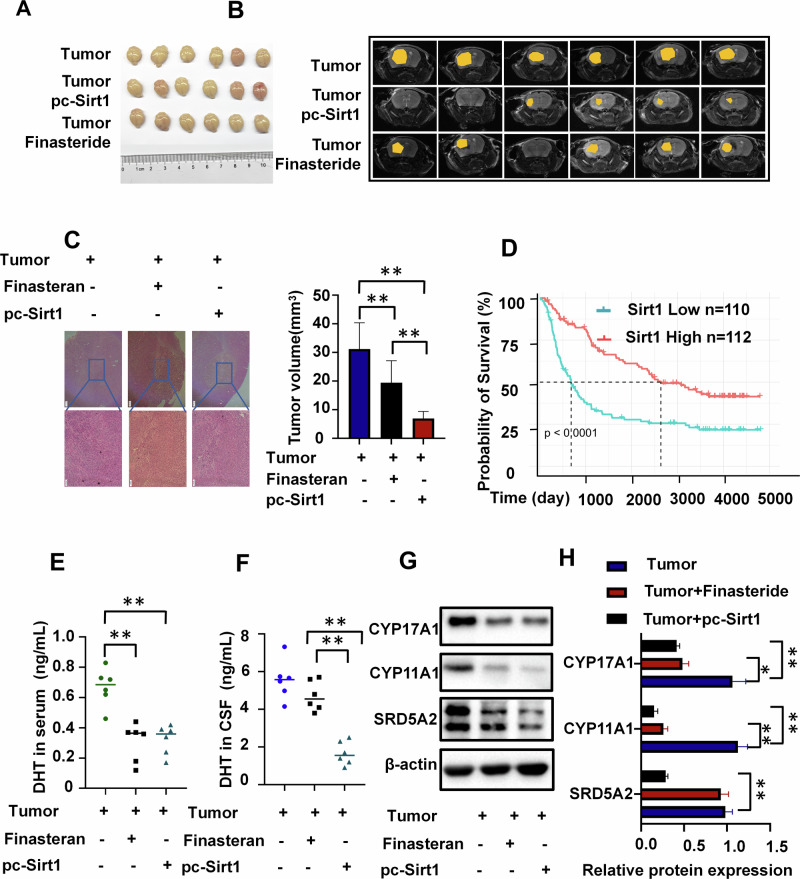
Effects of Sirt1 on DHT synthesis in GBM cells. **A** Animal tumor image (*n* = 6). **B, C** Tumor MRI of mice (**C**), H&E images and tumor volume of mice (*n* = 6), scale bars=200μm. **D** The Kaplan-Meier curve shows survival differences between GBM patients with high and low Sirt1 expression from Chinese Glioma Genome Atlas(CGGA) database(Sirt1 Low *n* = 110, Sirt1 High *n* = 112). **E, F** Testosterone levels in CSF and peripheral blood among three groups of mice: tumor control group, finasteride group, and Sirt1 overexpression group (*n* = 6). **G, H** Steroid enzyme levels in tumor cells among three groups of mice: tumor control group, finasteride group, and Sirt1 overexpression group (*n* = 3). *P < 0.05, **P < 0.01.

In animal experiments, comparing with the tumor group, the Sirt1 overexpression group showed the most pronounced efficacy in attenuating tumor progression, followed by the finasteride group and the control group (Fig. 6A~C). Notably, GBM patients with higher expression of Sirt1 had longer survival periods (Fig. 6D). Both Sirt1 overexpression and finasteride effectively reduced peripheral blood DHT levels in tumor-bearing mice(Fig. 6E). However, only Sirt1 overexpression significantly decreased DHT levels in the CSF of tumor-bearing mice(Fig. 6F). Additionally, both Sirt1 overexpression and finasteride reduced the levels of steroid enzyme in tumor tissue, with a more pronounced decrease observed in the Sirt1 overexpression group (Fig. 6G & H). The aforementioned results demonstrate that the overexpression of Sirt1 exerts a potent inhibitory effect on tumor cell secretion of androgens, thereby effectively delaying tumor progression.

## Discussion

Patients with GBM often exhibit elevated androgen levels, which promote tumor progression, yet the origin of these heightened androgens remains unclear. Our study uncovers that GBM cells themselves are a significant source of heightened androgen levels in both the CSF and peripheral blood of GBM patients. This self-secretion of androgen is attributed to the decreased expression of Sirt1 in GBM cells, which impairs its deacetylation of the transcription factor FOXO1. As a result, the increased transcriptional activity of FOXO1 induces the upregulation of steroid enzymes, consequently facilitating androgen production. Our findings provide crucial insights into the pathogenesis of GBM and highlight potential therapeutic targets for treatment.

Epidemiological studies have indicated a male predominance among glioma patients, with males exhibiting higher tumor progression rates compared to females [4, 6, 9, 39]. Elevated androgen levels have been linked to glioma progression and the development of drug resistance in GBM cells [40, 41]. Furthermore, glioma tissues often display heightened expression of the AR [9, 10]. Our research reveals a significant increase in testosterone levels in the CSF and peripheral blood of GBM patients. Additionally, as the pathological grade of glioma escalates, there is a corresponding elevation in androgen levels and their receptors, leading to a substantially reduced survival period. These findings underscore a positive role of androgen in GBM development, a notion supported by our in vitro experiments.

Recent research has explored reducing androgen levels to inhibit GBM progression and improve patient prognosis. Approaches include inhibiting the androgen receptor (AR) and using 5α-reductase inhibitors. However, AR mutations occur in over 30% of GBM cases, limiting the effectiveness AR-targeted therapies [42], 5α-reductase inhibitors can lower systemic dihydrotestosterone levels but may have adverse effects and may not effectively reduce androgen in CSF [43–45]. Our study shows short-term GBM progression inhibition by reducing exogenous androgen levels. However, prolonged reduction leads to upregulated steroid enzyme levels in GBM cells, causing fluctuating androgen levels and drug-resistance gene expression. Castration fails to reduce cerebrospinal fluid androgen levels and tumor progression in animal experiments, highlighting the need to identify the source of androgen within the brain in GBM patients.

Besides testicular interstitial cells, neurons and neuroglial cells can synthesize and express androgen, along with steroid enzymes such as P450c17, 3β-HSD, 17β-HSD, and P450 aromatase [46]. However, their expression patterns and activities differ in neurons and glial cells [47]. Our research revealed that GBM patients have higher testosterone levels in their CSF compared to peripheral blood, contrary to healthy individuals. Both GBM tissues and cells show a significant increase in steroid enzymes. After surgical resection of the tumor lesion, testosterone levels in the serum of glioma patients gradually decrease [7]. Interestingly, cholesterol, a precursor of androgen, is significantly increased in the cerebrospinal fluid of GBM patients, suggesting enhanced androgen synthesis capability in GBM cells. Our de-hormone culture of GBM cells has shown that U87 cells can synthesize androgen. Castration does not effectively reduce testosterone levels in the tumor group, indicating that the tumor is the primary factor influencing testosterone levels. Additionally, castration does not impact the level of androgen synthesis enzymes in GBM cells, while carcinogenesis upregulates their expression. Thus, tumor cells may the main source of high androgen levels in GBM patients.

The expression levels of Sirt1 in GBM have been debated, but its crucial role in various cancers is widely acknowledged [48–52]. In our study, high Sirt1 expression correlated with improved survival among GBM patients, suggesting its role as a tumor suppressor gene. Overexpression of Sirt1 suppressed androgen secretion in tumor cells and inhibited tumor progression. Sirt1 deacetylates both histone and non-histone proteins and is implicated in cancers [53]. FOXO1, involved in the development of various cancers, including hormone-dependent ones, influences androgen synthesis and regulates androgen receptor activity [54, 55]. We observed high FOXO1 protein expression in GBM, possibly due to post-translational modifications. Sirt1 overexpression effectively suppressed FOXO1 expression and reduced androgen secretion. In animal study, Sirt1 overexpression downregulated enzymes involved in androgen synthesis, exerting a significant anti-tumor effect.

## Conclusions

In summary, our research findings indicate that elevated androgen levels in the brains of GBM patients may originate from tumor cells through self-secretion. Additionally, our study suggests that Sirt1 acts as a tumor suppressor by promoting the deacetylation of FOXO1. By inhibiting the androgen synthesis process in tumor cells, this results in a decrease in androgen self-secretion, ultimately leading to tumor progression suppression. This study offers a comprehensive understanding of the pivotal role of Sirt1 in androgen self-secretion in GBM cells and underscores the potential of targeting Sirt1 as a therapeutic approach to inhibit androgen synthesis in GBM.

## Supplementary information

supplementary materials

## Data Availability

All scRNA-seq datasets are available online through the Gene Expression Omnibus (GEO) database https://www.ncbi.nlm.nih.gov/geo under the accession number GSE275600.
