# Supplementary material for: Suppression of FOXO1 activity by SIRT1-mediated deacetylation weakening the intratumoral androgen autocrine function in glioblastoma
Source: Cancer Gene Ther. 2025 Mar 12;32(3):343–54. doi: 10.1038/s41417-025-00880-1 (PMC11946903; doi:10.1038/s41417-025-00880-1)
Supplement: Supplementary file 1 — supplementary materials [file 41417_2025_880_MOESM1_ESM.pdf]

Supplementary Tables

Table S1. Antibodies

Antibody	Source	Identifier
AR	Cell Signaling Technology	5153S
β-actin	Servicebio	GB12001
CYP11A1	Cell Signaling Technology	12491
CYP17A1	Santa Cruz Biotechnology	sc-46081
GAPDH	Cell Signaling	2118
β-actin	Cell Signaling Technology	4967
SRD5A2	ABCAM	ab101896
NRG1	Santa	SC348
ABCB11	Sigma	MABS1193
BIRC3	Sigma	SAB1409216
FGF2	Sigma	SAB5700986
FOXO1	Sigma	SAB3500507
Tubulin	thermofisher	DM1A
Sirt1	Sigma	SAB5700048

Supplementary Figures

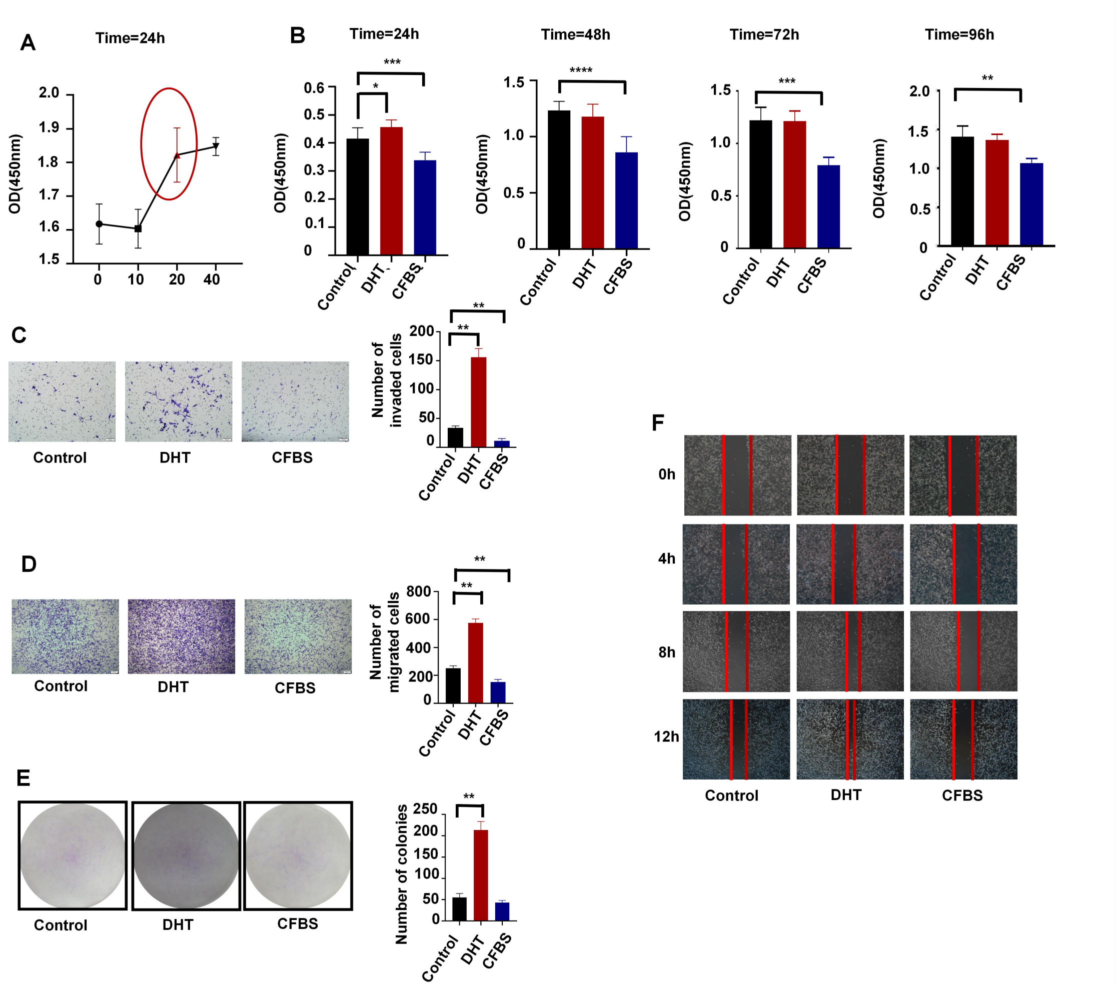

Figure S1 High levels of androgen promote the proliferation, invasion, migration of U87 cells. (A) The OD450 values of U87 cell cells treated with different concentrations of DHT for 24 hours. (B) The effects of DHT and CFBS on U87 cell viability at 24, 48, 72, and 96 h. (C) The effects of DHT and CFBS treatment on cell migration. (D) The effects of DHT and CFBS treatment for 12h on cell invasion. (E) The effects of DHT and CFBS treatment for 7 days on cell colony formation. (F) The effects of DHT and CFBS treatment on cell migration by wound healing assay. **P < 0.01, ***P＜0.001, ****P＜0.0001.

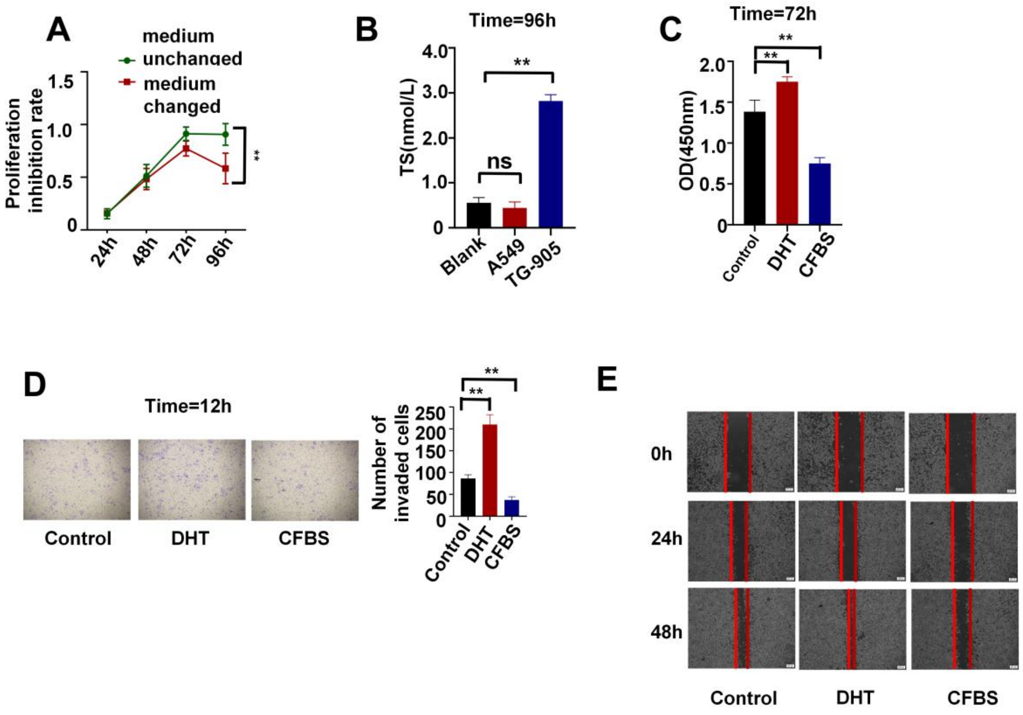

Figure 2S High levels of androgen promote the proliferation, invasion, migration of GBM (TG-905) cells. (A) Proliferation inhibition curve of TG-905 cells under charcoal-stripped fetal bovine serum(CFBS) culture (24h medium change vs continuous medium retention , n=3). (B) Androgen concentration in a blank control medium , the culture medium of A549(negative control) and TG-905 cells cultured for 96h without androgen (continuous medium retention, n=3). (C) The OD450 values of TG-905 cell cells treated with different concentrations of DHT for 72 hours. (D) The effects of DHT and CFBS treatment for 12h on cell invasion(n=3). (E) The effects of DHT and CFBS treatment on cell migration by wound healing assay(n=3), scale bars=200μm. **P＜0.01.
